# Sarcoidosis Associated Pulmonary Hypertension

**DOI:** 10.3390/biomedicines12010177

**Published:** 2024-01-13

**Authors:** Alexander Liu, Laura C. Price, Rakesh Sharma, Athol U. Wells, Vasileios Kouranos

**Affiliations:** Royal Brompton Hospital, Part of Guy’s and St. Thomas’ NHS Foundation Trust, London SW3 6NP, UK; a.liu@rbht.nhs.uk (A.L.); laura.price@rbht.nhs.uk (L.C.P.); rakesh.sharma@rbht.nhs.uk (R.S.); rbhild@rbht.nhs.uk (A.U.W.)

**Keywords:** sarcoidosis, pulmonary hypertension, pulmonary sarcoidosis, cardiac sarcoidosis

## Abstract

In patients with sarcoidosis, the development of pulmonary hypertension is associated with significant morbidity and mortality. The global prevalence of sarcoidosis-associated pulmonary hypertension (SAPH) reportedly ranges between 2.9% and 20% of sarcoidosis patients. Multiple factors may contribute to the development of SAPH, including advanced parenchymal lung disease, severe systolic and/or diastolic left ventricular dysfunction, veno-occlusive or thromboembolic disease, as well as extrinsic factors such as pulmonary vascular compression from enlarged lymph nodes, anemia, and liver disease. Early diagnosis of SAPH is important but rarely achieved primarily due to insufficiently accurate screening strategies, which rely entirely on non-invasive tests and clinical assessment. The definitive diagnosis of SAPH requires right heart catheterization (RHC), with transthoracic echocardiography as the recommended gatekeeper to RHC according to current guidelines. A 6-min walk test (6MWT) had the greatest prognostic value in SAPH patients based on recent registry outcomes, while advanced lung disease determined using a reduced D_LCO_ (<35% predicted) was associated with reduced transplant-free survival in pre-capillary SAPH. Clinical management involves the identification and treatment of the underlying mechanism. Pulmonary vasodilators are useful in several scenarios, especially when a pulmonary vascular phenotype predominates. End-stage SAPH may warrant consideration for lung transplantation, which remains a high-risk option. Multi-centered randomized controlled trials are required to develop existing therapies further and improve the prognosis of SAPH patients.

## 1. Introduction

Sarcoidosis is a multisystem inflammatory condition characterized by the formation of non-caseating granulomas in the affected tissues [1]. Pulmonary involvement occurs in up to 90% of patients with sarcoidosis, which can affect the parenchyma, the lymph nodes, and the airways [2,3]. The development of sarcoidosis-associated pulmonary hypertension (SAPH) is associated with impaired functional capacity and a significant risk of morbidity and mortality [4]. The pathophysiological processes underlying SAPH can be multifactorial, which form the basis for variations in management strategies [5]. The treatment options for SAPH rely predominantly on those developed for non-sarcoid patients with pulmonary hypertension (PH) and the optimization of underlying risk factors [6,7]. In this review, we will discuss the pathophysiological basis for SAPH as well as outline the current screening, diagnostic, and treatment strategies.

## 2. Definitions of PH in Sarcoidosis

The definitive diagnosis of PH requires the measurement of hemodynamic parameters during right heart catheterization (RHC) [6]. PH is currently defined as an elevated mean pulmonary arterial pressure (mPAP) of greater than 20 mmHg [6]. The threshold of mPAP associated with PH was reduced in the recent European Society of Cardiology (ESC) and the European Respiratory Society (ERS) guidelines [6] (from 25 mmHg previously), as supported by clinical evidence of the upper limit of normal pulmonary arterial pressures in healthy individuals [6,8,9] and findings from studies demonstrating the prognostic significance of mildly elevated pulmonary arteries pressures [6,10,11,12]. The classical system of further categorizing PH into pre-capillary PH, post-capillary PH, or combined pre- and post-capillary PH remains [6].

**Figure 1:**
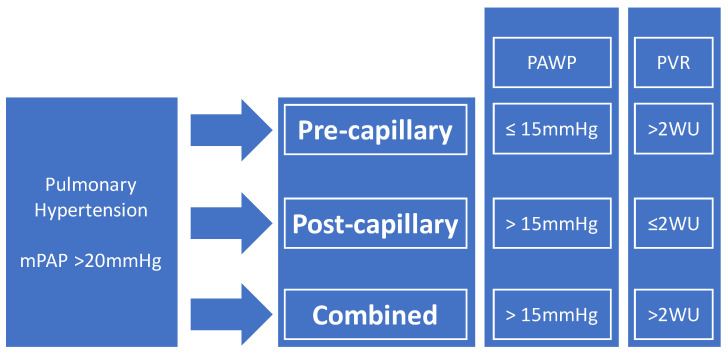
Definition of pulmonary hypertension (PH). mPAP: mean pulmonary arterial pressure; PAWP: pulmonary arterial wedge pressure; PVR: pulmonary vascular resistance; WU: wood units.

Pre-capillary PH is defined as mPAP > 20 mmHg with pulmonary arterial wedge pressure (PAWP) ≤ 15 mmHg and pulmonary vascular resistance (PVR) > 2 Wood Units (WU) [6]. Post-capillary PH is defined as mPAP > 20 mmHg with PAWP > 15 mmHg and PVR ≤ 2 WU [6]. Combined pre- and post-capillary PH is defined as mPAP > 20 mmHg with PAWP > 15 mmHg and PVR > 2 WU [6] (Figure 1).

## 3. Epidemiology

Single-centered epidemiological studies on SAPH have shown a diverse range of estimated SAPH prevalence, ranging from 3.6% to greater than 20% [13,14]. The PULSAR study included 399 sarcoidosis patients, in whom the prevalence of SAPH was reported to be around 2.9% [15]. SAPH is more prevalent in patients with advanced sarcoidosis, reported to be as high as 62% [16], including those being considered for lung transplantation [17]. It must be noted that the prevalence of PH reported to date includes patients with mPAP > 25 mmHg in RHC. As a result, it is expected that these figures would increase if patients with milder disease are added. This becomes very important in the general sarcoidosis population, where there is currently no validated definition of the PH suspected population. A significant number of patients with systemic sarcoidosis would report exertional breathlessness in a clinic setting, but very few of them will be found to have PH. Appropriate screening strategies are required to detect this population better.

The international, multi-centered Registry for Sarcoidosis Associated Pulmonary Hypertension (ReSAPH) showed in 176 patients with right heart catheterization confirmed pre-capillary SAPH that clinical features of SAPH were similar across multiple centers in the US, Europe, and the Middle East [18]. There was a greater female representation in the registry population [18]. The severity of SAPH was found to be related to reduced diffusing capacity for carbon monoxide (D_LCO_) [18]. The registry also identified a significantly higher rate of treatment in non-US centers compared with US centers [18]. The multi-center French registry showed in 126 SAPH patients that 54% were on long-term oxygen therapy over a 10-year follow-up period, and 83% of patients had significant symptoms in World Health Organisation (WHO) functional classes (FC) III–IV [18].

## 4. Pathophysiology

SAPH remains in the miscellaneous group of PH based on the World Health Organisation (WHO) PH classification because multiple pathophysiological mechanisms can lead to its development. Specifically, SAPH can develop in relation to (a) advanced parenchymal lung disease, (b) systolic/diastolic LV dysfunction in the form of cardiac sarcoidosis or alternative underlying cardiac disease, (c) veno-occlusive disease reported in patients with sarcoidosis [19,20], (d) thromboembolic disease that has been found to have a higher prevalence in sarcoidosis patients compared with non-sarcoid subjects [21], (e) extrinsic pulmonary vascular compression from mediastinal lymph nodes [22,23,24] and (f) direct sarcoidosis induced vascular granulomatous inflammation [25,26]. Furthermore, conditions such as obstructive sleep apnoea (OSA), anemia, and chronic liver disease are known contributors to increased pulmonary vascular resistance, irrespective of the underlying sarcoidosis diagnosis [27,28,29].

### 4.1. Parenchymal Lung Disease

SAPH is more prevalent in patients with fibrotic lung disease, whereby alveolar capillary disruption, ensuing fibrotic replacement, and chronic hypoxia contribute to the development of pre-capillary PH [17]. However, the severity of PH does not always appear to be associated with the degree or extent of lung fibrosis in SAPH patients, raising the suspicion of concomitant vascular inflammation [5].

In the ReSAPH registry, the mean D_LCO_ levels were a predicted 40% and a predicted FVC of 62% [30], suggesting a predominance of fibrotic lung disease [18,30]. Scadding stage IV lung disease was observed on chest radiographs in approximately 66% of SAPH patients [30]. Further studies evaluating the extent of pulmonary fibrosis using computed tomography (CT) are required to ascertain the significance of this observation.

### 4.2. Cardiac Diseases

Left-sided cardiac disease in sarcoidosis patients can be due to either cardiac involvement of sarcoidosis and/or concomitant non-sarcoid etiologies such as ischemic cardiomyopathy or valvular heart disease [7]. Left-sided cardiac diseases predominantly lead to post-capillary or combined SAPH [7].

Cardiac involvement occurs in around a quarter of patients with sarcoidosis and can manifest in the development of heart failure, ventricular arrhythmias, high-grade atrioventricular blocks, or be diagnosed at post-mortem [31]. Both left ventricular (LV) systolic and diastolic dysfunction have been reported in patients with cardiac sarcoidosis [31]. Increased LV filling pressures and the resultant retrograde pressure transmission through the pulmonary vasculature can be detected as elevated PAWP during RHC, which is a feature of post-capillary PH [7]. The increased pulmonary vascular pressures can also lead to chronic pulmonary vasoconstriction, elevated PVR, and pre-capillary PH [32]. It is important to note that SAPH associated with LV dysfunction appears to have a better outcome compared with those with preserved LV function [33].

Right ventricular (RV) dysfunction is usually a secondary phenomenon associated with SAPH, and isolated RV dysfunction as the cause of SAPH is rare [5,7,34]. Cardiac involvement of the RV (isolated or in combination with the LV) has been reported in the sarcoidosis population [34]. However, in most cases, systemic volume congestion and overload as a result of LV dysfunction in the presence or absence of advanced parenchymal lung disease are the most common contributors to the development of RV dysfunction and SAPH [5,7,31]. Optimal immunosuppressive treatment in the case of RV involvement may be beneficial for SAPH features in certain patients, highlighting the importance of a multidisciplinary approach in the management of SAPH patients.

### 4.3. Veno-Occlusive Disease

Veno-occlusive SAPH can be driven by the formation of non-caseating granulomas in the pulmonary vasculature [26]. Granulomatous involvement can affect large pulmonary arteries as well as small venules [26]. Although histologically distinct in locations, little clinical data suggests different responses to treatment in patients with granulomatous involvement in arterial vs. venous systems, which deserves further investigation. The treatment of these patients is largely based on clinical experience. Patients with large pulmonary artery involvement are expected to have a greater response to immunosuppression, but no definitive data are available to support that.

Granulomatous infiltration can be extensive, affecting the vascular wall in a circumferential and transmural fashion, involving the entire pulmonary vascular tree from the elastic arteries to the venules [26]. All layers of the vessel wall can be affected, leading to replacement fibrosis [26]. Granulomas can also be found in the pulmonary lymphatic vessels, as well as the vasa vasorum [26].

In specific cases, granulomatous inflammation of the pulmonary vasculature and the lymphatics can result in a pulmonary veno-occlusive phenomenon characterized by elevated PVR and usually post-capillary PH [5,7,26].

### 4.4. Thromboembolic Disease

In population-based studies, sarcoidosis is associated with a 2-3-fold increased risk of venous thromboembolism (VTE) [35,36,37]. While the exact cause of this observation is unclear, it may be related to an inflammation-driven systemic hypercoagulable state often encountered in patients with sarcoidosis [7,36,37]. As a result, SAPH patients can develop large, central pulmonary emboli as well as smaller, segmental, or sub-segmental emboli [7].

### 4.5. Extrinsic Compressors and Other Factors

**Figure 2:**
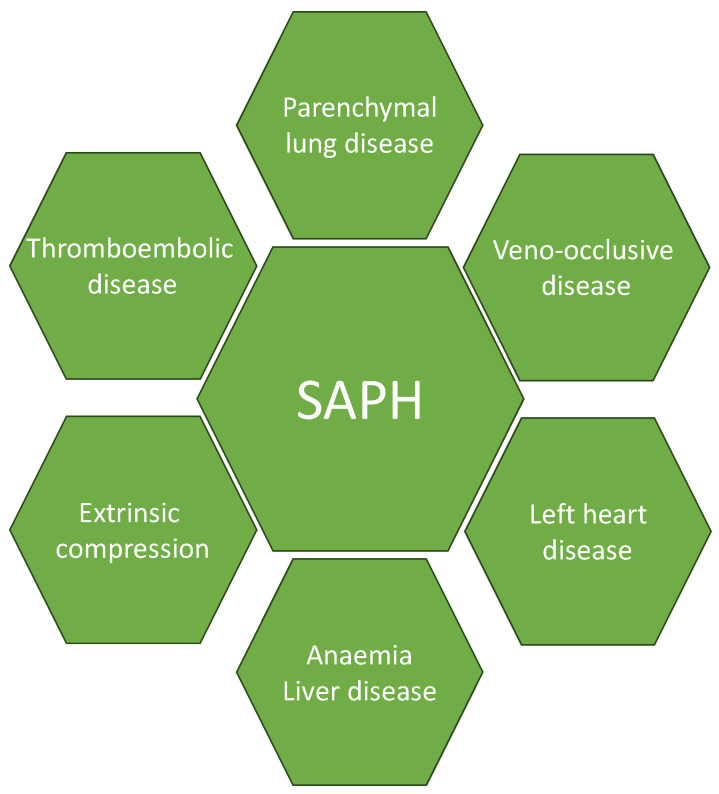
Mechanisms of sarcoidosis associated with pulmonary hypertension (SAPH).

External compression of pulmonary vessels by large hilar and mediastinal lymph nodes can lead to luminal stenoses, restricted blood flow, and PH [5,7,23]. Anemia may be associated with high-output cardiac failure, and chronic liver disease can lead to portal–systemic congestion; these may contribute to the pathogenesis of SAPH in some cases [7]. Obstructive sleep apnoea (OSA) also occurs more commonly in patients with sarcoidosis, particularly when associated with weight gain related to corticosteroid therapy, which can lead to SAPH [7]. The pathophysiological mechanisms of SAPH are illustrated in Figure 2.

## 5. Screening and Diagnosis

Currently, one of the most difficult challenges in the management of SAPH lies in establishing a timely diagnosis [30,38]. Contemporary evidence suggests that after the first diagnosis of sarcoidosis, it can take up to 12–17 years for a subsequent SAPH diagnosis to be reached [30,38]. In most patients in whom SAPH is first diagnosed, the degree of PH tends to be already severe, suggesting that the diagnosis is made at a late stage in the disease process [5].

In the absence of accurate and validated screening methods for SAPH, all available clinical information needs to be considered. Current guidelines recommend the performance of echocardiography as a gatekeeper to the performance of RHC for invasive hemodynamic measurements that ultimately offer a definitive diagnosis [39]. Nonetheless, there have been several studies showing discrepancies between mPAP measurements on RHC and estimated PASP measurements on echocardiography [39].

We would strongly recommend the consideration of the following parameters when suspecting SAPH: (a) an electrocardiogram (ECG) for features of PH; (b) lung function tests (predominantly reductions in D_LCO_ levels disproportionate to lung volumes, as judged by Kco levels or FVC/D_LCO_ ratios); (c) Alveolar-arterial (A-a) gradient; (d) chest CT scan abnormalities indicative of SAPH (MPA/AA diameter ratio); (e) serum B-type natriuretic peptide (BNP) or N-terminal Pro-BNP (NT-proBNP) levels; (f) desaturation in 6-min walk tests (6MWTs) and the 6MWT distance; (g) RV dysfunction on cardiovascular magnetic resonance (CMR) imaging; and (h) echocardiography to estimate probability of PH (Table 1).

### 5.1. Clinical Symptoms and Signs

Symptoms such as exertional breathlessness, chest discomfort, and palpitations are not specific to SAPH; rather, they can also be due to pulmonary sarcoidosis and/or cardiovascular diseases [7]. Syncope is rare but a concerning feature, which should prompt screening for both SAPH and heart blocks and/or ventricular arrhythmias associated with cardiac sarcoidosis [6,31]. A deterioration in symptomology or the advent of symptoms consistent with congestive heart failure in previously stable sarcoid patients should prompt the consideration of SAPH as a possible cause [5,7].

Clinical signs in SAPH may be indistinguishable from those in PH due to non-sarcoid disease [7]. These include features of RV failure, such as a loud pulmonary component of the second heart sound (P2), elevated jugular venous pressure (JVP), and peripheral edema [7]. Signs of RV hypertrophy or strain, such as precordial RV heave, may also be present [7]. The presence of these signs indicates that SAPH may have progressed to a late stage, with possible evidence of RV remodeling [7].

### 5.2. Electrocardiogram (ECG)

ECG is a relatively simple bedside test to perform in the clinical setting. Although features such as p-wave pulmonale, right axis deviation, right bundle branch block, and RV strain can occur in PH [6], these are not specific to SAPH. According to the WASOG statement, identifying ECG abnormalities suggestive of PH forms part of the initial assessment of SAPH patients, along with clinical signs, circulating biomarkers, and imaging parameters [39].

### 5.3. Chest X-ray (CXR) and CT

CXR findings can reflect the presence of lung parenchymal disease associated with SAPH [39]. In SAPH patients without significant parenchymal disease, CXR may be useful for surveillance [7]. Abnormal CXR findings should be further characterized by CT imaging. Although a simple test, there is currently no established role for the use of CXR in either screening or diagnosis of SAPH.

CT forms part of the first-line imaging workup for SAPH [7]. Increased main pulmonary artery (MPA) diameter in relation to the ascending aortic (AA) diameter (MPA/AA) and RV dilatation on CT are correlated with the presence of PH [40,41]. CT pulmonary angiography also enables the detection of central and segmental pulmonary emboli [42]. CT also enables a one-stop assessment of lung parenchymal disease and the degree of pulmonary fibrosis [5]. Although CT cannot provide a definitive diagnosis of SAPH, it enables imaging surveillance of lung parenchymal pathology to help gauge clinical vigilance for further investigation of SAPH.

### 5.4. Functional Tests

A pulmonary function test (PFT) tends to be performed early after a patient is diagnosed with sarcoidosis and offers an ideal opportunity to screen for SAPH [5]. Forced vital capacity (FVC) and diffusing capacity of the lung for carbon monoxide (D_LCO_) is reduced disproportionately to lung volume in patients with SAPH [7,43,44].

The 6-min walk test (6MWT) distance provides non-invasive screening for reduced functional capacity in SAPH [5]. Sarcoidosis patients who experience oxygen desaturations to less than 90% in the 6MWT have a 12-fold elevated risk of having SAPH [5]. The International Registry of Sarcoidosis Associated Pulmonary Hypertension (ReSAPH) showed combined reductions in D_LCO_ (<35% predicted) and the 6MWT distance (<300 m) are linked to reduced transplant-free survival in patients with SAPH [30]. A preserved FEV_1_/FVC ratio was found to be an independent risk factor for worsened outcomes in SAPH patients [30]. These ReSAPH data also indicated that the 6MWD had the greatest prognostic value in SAPH, which correlated with other physiologic and hemodynamic variables [45].

### 5.5. Ventilation/Perfusion (V/Q) Scan

Although the role of VQ scans for assessing ventilation/perfusion mismatch is established in the workup for chronic thromboembolic PH (CTEPH) [6], its utility for the assessment of SAPH is less well characterized [19,46]. In a small retrospective series of eight patients with either radiological (8/8) or histological (7/8) diagnosis of sarcoidosis and CTEPH, 50% (4/8) of patients had evidence of V/Q mismatch, and slightly more (63%; 5/8) patients had CTPA evidence supportive of CTEPH [46]. In other case reports of patients with sarcoidosis, V/Q scans have also been used to assess the likelihood of CTEPH [19].

### 5.6. Transthoracic Echocardiography (TTE)

The use of TTE for PH screening is well-established in clinical guidelines [6]. TTE is also particularly useful in sarcoidosis patients as a screening tool for cardiac involvement [31]. TTE uses the tricuspid regurgitation (TR) velocity and the inferior vena cava status to estimate the pulmonary arterial systolic pressures (PASP). The PASP and other TTE features suggestive of PH are used in combination to estimate the probability of PH [6].

Although commonly used in clinical practice, TTE has several limitations. Prominent TR jets can be absent, which prevents the estimation of PASP [7]. Further, PASP, as estimated using TTE, is unreliable in patients with significant pulmonary fibrosis [5]. Diagnostic imaging on TTE also relies on adequate transthoracic windows, which are not always present in patients with lung disease [7]. There is little evidence supporting the use of TTE for PH screening in asymptomatic patients with sarcoidosis.

### 5.7. Cardiovascular Magnetic Resonance (CMR) and Positron Emission Tomography (PET)

CMR provides a multi-parametric assessment of cardiac volumes, systolic function, and myocardial tissue characterization [47]. CMR offers a detailed assessment of RV size and systolic function, as well as measurements for PA and aortic diameters, which act as surrogate markers for PH [47]. CMR also forms part of the advanced imaging workup for cardiac sarcoidosis [31], while late gadolinium enhancement (LGE) imaging enables the assessment of myocardial fibrosis and potential etiology of cardiac dysfunction [47]. Despite its advantages, CMR is not routinely used for the direct assessment of SAPH beyond the detection of cardiac involvement of sarcoidosis.

The 18F-fluorodeoxyglucose (FDG)-PET-CT technique enables the detection of myocardial inflammation in the clinical workup for cardiac sarcoidosis [31]. Uptake of FDG in the PA and RV have also been found in patients with pulmonary arterial hypertension (PAH) and RV failure, indicating the possible presence of active metabolism or inflammation. However, the increased intraventricular pressures could result in similar appearances [48,49]. Therefore, it remains currently unclear whether this reported FDG uptake is due to pulmonary hypertension itself and/or inflammation related to vasculitis. Based on our clinical experience, we tend to avoid treating patients with SAPH with immunosuppression in isolation on the basis of this finding unless there is multifocal FDG uptake in the myocardium suggesting cardiac involvement.

FDG-PET-CT may also serve a role in disease monitoring and in assessing the response to PH-directed therapies [50,51,52,53]. These potential indications require further investigation, and the clinical role of FDG-PET-CT in SAPH assessment remains focused on the assessment of cardiac sarcoidosis.

### 5.8. Invasive Assessment

**Figure 3:**
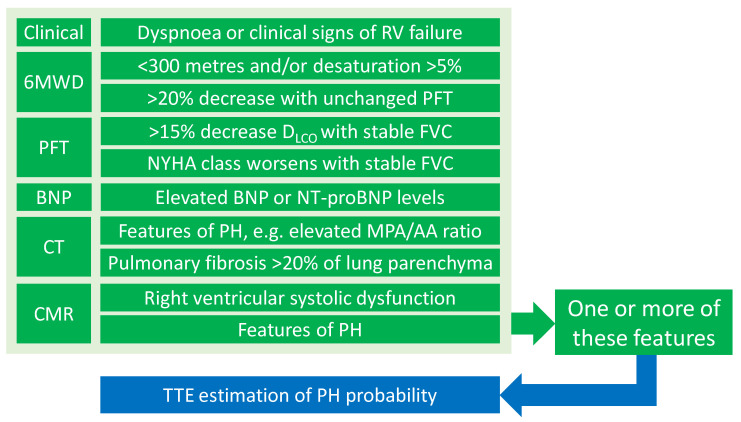
An assessment algorithm for sarcoidosis-associated pulmonary hypertension based on the WASOG statement [39]. The 6MWD: 6-min walk distance; AA: ascending aorta; BNP: B-type natriuretic peptide; CMR: cardiovascular magnetic resonance; CT: computed tomography; CXR: chest X-ray; D_LCO_: diffusing capacity for carbon monoxide; FVC: forced vital capacity; MPA: main pulmonary artery; NT-proBNP: N-terminal Pro-BNP; NYHA: New York Heart Association; PFT: pulmonary function tests; RV: right ventricular; PH: pulmonary hypertension; TTE: transthoracic echocardiogram.

RHC provides the definitive diagnosis of SAPH and should be considered in patients with sarcoidosis and suspected PH from non-invasive screening [5]. RHC is best avoided in patients with decompensated heart failure or chest infections until these have been treated [7]. Furthermore, routine vaso-reactivity testing during RHC is not supported by the current evidence since the degree of responsiveness to inhaled nitric oxide is a poor predictor of therapeutic response with sildenafil and calcium channel antagonists [5,54,55]. Figure 3 illustrates the screening and diagnostic modalities for SAPH.

### 5.9. Multidisciplinary Approach in the Diagnosis and Management

In view of (a) the complexity of various pathogenetic mechanisms and (b) the multisystem nature of sarcoidosis, our group strongly supports the role of a multidisciplinary approach to the diagnosis and management of SAPH. Such an approach incorporating all the available clinical information, imaging, histopathology, and right heart catheterization data can provide a more accurate diagnosis and patient-centered treatment plan. The lack of communication between healthcare professionals can lead to fragmented care and poor diagnostic and treatment outcomes. For example, the role of immunosuppression in the context of SAPH has not yet been well defined but has been found to be helpful in the appropriate clinical context. In addition, our clinical experience suggests that pulmonary vasodilators have a clear role in stabilizing and even improving certain patients with SAPH on a case-to-case basis. Therefore, the coordination of both approaches remains cardinal in the management of such patients.

## 6. Clinical Management

Management of SAPH should take into account the mechanistic cause of the PH, the severity of both the PH and the underlying parenchymal lung disease [39]. Therapeutic decisions should be made by multidisciplinary teams, including PH experts [39].

Supportive management, such as oxygen therapy, can be helpful in the presence of resting hypoxemia [56], and exercise programs used in non-sarcoidosis PH patients can also be considered in patients with SAPH [57,58]. However, these therapies have little prognostic data in SAPH.

The WASOG recommends that PAH therapy for symptomatic patients with pre-capillary SAPH should be considered on a case-by-case basis [39], which remains an off-label use. Therapy for pre-capillary hypertension is directed towards treating the underlying vascular disease, and care should be taken when using PAH therapies in patients with post-capillary PH, such as pulmonary veno-occlusive disease and PH due to left-sided heart disease [39]. Furthermore, PAH therapies may be less effective in patients with moderate-to-severe parenchymal lung disease but may still be indicated in patients with evidence of RV systolic dysfunction [39].

In patients with left-sided cardiac disease, consideration should be given to the workup and treatment of cardiac sarcoidosis and LV dysfunction, as SAPH-targeted therapies have been found to have no beneficial effect and may even be harmful [59]. Treatment aimed at improving hypoxia and hypercapnia may be helpful in patients with SAPH [5].

Patients with chronic thromboembolic disease warrant both medical and interventional management, including pulmonary endarterectomy or balloon angioplasty [5]. Stenting of the pulmonary arteries or veins can help to relieve external vascular constriction [22,24], and treatment of co-morbidities such as anemia and chronic liver disease can also be therapeutically beneficial [5,7].

Timely referral to a specialist center with experience in managing SAPH patients can enable expert evaluation, minimizing any delays in reaching a diagnosis and initiating therapy. In complex cases where more than one type of PH is present, for instance, patients with pulmonary vascular and parenchymal disease, management guided by consensus decisions reached by a multidisciplinary team (MDT) meeting may be particularly beneficial.

In patients with end-stage SAPH, lung transplantation can be considered [60], although this needs to be reviewed with caution and on a case-by-case basis, as several phenotypes have emerged [60]. The option of heart–lung transplantation has been reported in some cases [61,62]. PH is not only prevalent in patients listed for lung transplantation [60]; lung transplantation candidates with SAPH are also known to require more supplementary oxygen than those without SAPH [17,39]. The long-term outcome of SAPH patients after lung transplantation may be similar to those with non-sarcoid indications [60,63]; however, most studies on this topic have focused on the post-transplantation survival assessment of sarcoidosis patients as a whole rather than specifically on patients with SAPH [60,63]. Le Pavec and colleagues showed that in 112 sarcoidosis patients post-lung transplantation, advanced age and extensive pre-operative pulmonary fibrosis were predictors of mortality [60]. In the same study, neither mean pulmonary arterial pressure nor pulmonary arterial wedge pressure were found to be significant predictors of mortality in post-transplant patients [60]. This suggests that the invasively measured PH severity did not have a significant prognostic influence in these patients [60], which requires further investigation. There is currently limited evidence supporting the exclusion of sarcoidosis patients with severe PH from being considered for lung transplantations, which is considered on a case-to-case basis.

The psychological impact of PH on patients is an important area to consider [64]. Indeed, the prevalence of anxiety and depression has been shown to be up to 50% in PH patients [65]. Recent evidence suggests a potential role for psychotherapy in the management of anxiety and depression in PH patients [66], which needs to be validated in larger studies [66]. Studies have also sought to develop assessment tools to facilitate the evaluation of the psychological impact of symptoms in PH patients [67,68]. There is currently a paucity of studies specifically focusing on the psychological impact of SAPH patients. Although psychological and palliative care support is expected to positively impact therapy compliance, quality of life, and overall clinical management, research is required in SAPH patients to confirm this hypothesis.

## 7. Drug Treatments of SAPH

Up to 77% of patients with SAPH are treated with pulmonary vasodilators [5,30]. However, the evidence supporting their use in SAPH is mostly based on small-scale studies [69]. In the UK, pulmonary vasodilator therapy is indicated in certain SAPH patients, while such use is off-label in other countries [5]. Pulmonary vasodilator therapy has been linked to improved hemodynamic and functional parameters, with little dependence on the presence of lung fibrosis [5,7]. However, existing studies have failed to demonstrate a prognostic benefit with such treatments in reducing mortality in SAPH patients [5,7].

### 7.1. Endothelin Receptor Antagonists

In a double-blind, randomized controlled trial, mPAP and PVR were reduced significantly in SAPH patients treated with bosentan (*n* = 23; up to 125 mg) but not in those treated with placebo (*n* = 12) [69]. However, the 6MWT did not alter significantly in either treatment arm [69]. In a prospective, open-label, proof-of-concept trial of 21 SAPH patients, treatment with ambrisentan was not associated with significant improvements in 6WMT, quality of life, or B-type natriuretic peptide (BNP) levels [70]. There was a high dropout rate of 52% in the study [70]. In a retrospective study of six patients with severe SAPH treated with macitentan (with sildenafil in four patients), functional capacity improved in four patients [71].

### 7.2. Phosphodiesterase 5 Inhibitors (PDE5i)

The evidence behind the clinical use of PDE5i in SAPH is mainly based on retrospective data [72,73,74]. In a single-centered study, sildenafil monotherapy in 25 patients with end-stage pulmonary sarcoidosis was linked with reduced mPAP and PVR, improved cardiac output, and index; the 6MWT did not change significantly [72]. When used as part of combination therapy, sildenafil was associated with improvements in the 6MWT, BNP levels, and echocardiographic TR severity [74]. Tadalafil therapy has failed to demonstrate improvements in the 6MWT or BNP levels and has a high drop rate from therapy [73].

### 7.3. Soluble Guanylate Cyclase Stimulator

In a recent double-blind placebo-controlled trial, riociguat was well tolerated [75]. The primary endpoint was reached in five out of eight placebo-treated patients and in none of the eight riociguat-treated patients [75]. The primary endpoint included all-cause mortality, hospitalization due to worsening cardiopulmonary status attributable to the progression of the disease, >50 m decrease in the 6MWD test, or worsening of World Health Organization functional class [75]. The 6MWT distance also improved in the riociguat-treated group but worsened in the placebo group [75].

### 7.4. Prostacyclin-Based Therapy

In SAPH patients, prostacyclin therapy has been associated with improvements in functional capacity, BNP levels, and hemodynamic parameters [76]. Inhaled iloprost has been shown to lead to reduced mPAP and PVR with improvements in quality of life [77]. Previous reports of worsening oxygenation during the acute stage of prostacyclin-based therapy have been shown to be less apparent in recent studies [5].

### 7.5. Combination Therapy

The clinical evidence on the use of combination vasodilator therapies is limited [78,79]. In a study of three patients with SAPH, combination therapy with ambrisentan and tadalafil led to hemodynamic and functional improvements [78]. Sequential therapy with macitentan followed by tadalafil was also associated with improvements in hemodynamic parameters, exercise capacity, and functional class in a case report of one patient with SAPH [79].

### 7.6. Immunosuppressive Therapy

There is currently little evidence directly linking immunosuppressive therapy to improvements in hemodynamic parameters or functional capacity in SAPH [7,38]. The main utility of immunosuppressive therapy appears to be in combination with pulmonary vasodilator drugs for the management of patients with SAPH [18]. There is little evidence supporting the prognostic impact of steroid therapy in SAPH patients [20].

However, the exact role of immunosuppressive therapy in the management of SAPH is not yet well defined. The decision is made on a case-by-case basis, and the use of a multidisciplinary team (MDT) approach to the management of SAPH patients is strongly recommended. Given the fact that such a diagnosis is reached late in the disease evolution, it is highly likely that the patients are already on immunosuppressive treatment at the time of diagnosis. Patients with active advanced pulmonary disease and/or active cardiac disease with concomitant SAPH would definitely benefit from a trial of immunosuppressive treatment escalation, depending on the clinical scenario.

## 8. Conclusions

SAPH is associated with significant morbidity and mortality. Achieving early diagnosis of SAPH is challenging, and a range of screening tests are available. The treatment of SAPH is dependent on the underlying mechanism, which involves medical and procedural therapies. Further work is needed to better understand the epidemiology and therapeutic strategies for SAPH.

## Figures and Tables

**Table 1:** Salient investigations for suspected SAPH.

Investigations	Salient Parameters
Electrocardiogram	Features of PH
Lung function test	Disproportionate reduction in D_LCO_; Kco
Six-minute walk test	Six-minute walk distance and desaturation
Arterial blood gas sample	Alveolar-arterial gradient
Blood test sample	BNP or NT-proBNP
Chest CT	MPA/AA diameter ratio, pulmonary fibrosis
CMR	RV dysfunction and PH features
Echocardiography	Probability of pulmonary hypertension

AA: ascending aorta; BNP: B-type natriuretic peptide; CMR: cardiovascular magnetic resonance; CT: computed tomography; D_LCO_: diffusing capacity for carbon monoxide; Kco: carbon monoxide transfer coefficient; MPA: main pulmonary artery; NT-proBNP: N-terminal Pro-BNP; PH: pulmonary hypertension; RV: right ventricular.

## Data Availability

Not applicable.

## References

[1] Churg A., Carrington C.B., Gupta R. (1979). Necrotizing Sarcoid Granulomatosis. Chest.

[2] Baughman R.P., Teirstein A.S., Judson M.A., Rossman M.D., Yeager H., Bresnitz E.A., DePalo L., Hunninghake G., Iannuzzi M.C., Johns C.J. (2001). Clinical Characteristics of Patients in a Case Control Study of Sarcoidosis. Am. J. Respir. Crit. Care Med..

[3] Jain R., Yadav D., Puranik N., Guleria R., Jin J.O. (2020). Sarcoidosis: Causes, Diagnosis, Clinical Features, and Treatments. J. Clin. Med..

[4] Diaz-Guzman E., Farver C., Parambil J., Culver D.A. (2008). Pulmonary hypertension caused by sarcoidosis. Clin. Chest Med..

[5] Samaranayake C.B., McCabe C., Wort S.J., Price L.C. (2021). Sarcoidosis associated pulmonary hypertension: An update. Curr. Opin. Pulm. Med..

[6] Humbert M., Kovacs G., Hoeper M.M., Badagliacca R., Berger R.M.F., Brida M., Carlsen J., Coats A.J.S., Escribano-Subias P., Ferrari P. (2022). 2022 ESC/ERS Guidelines for the diagnosis and treatment of pulmonary hypertension: Developed by the task force for the diagnosis and treatment of pulmonary hypertension of the European Society of Cardiology (ESC) and the European Respiratory Society (ERS). Endorsed by the International Society for Heart and Lung Transplantation (ISHLT) and the European Reference Network on rare respiratory diseases (ERN-LUNG). Eur. Heart J..

[7] Duong H., Bonham C.A. (2018). Sarcoidosis-associated Pulmonary Hypertension: Pathophysiology, Diagnosis, and Treatment. Clin. Pulm. Med..

[8] Kovacs G., Berghold A., Scheidl S., Olschewski H. (2009). Pulmonary arterial pressure during rest and exercise in healthy subjects: A systematic review. Eur. Respir. J..

[9] Kovacs G., Olschewski A., Berghold A., Olschewski H. (2012). Pulmonary vascular resistances during exercise in normal subjects: A systematic review. Eur. Respir. J..

[10] Maron B.A., Hess E., Maddox T.M., Opotowsky A.R., Tedford R.J., Lahm T., Joynt K.E., Kass D.J., Stephens T., Stanislawski M.A. (2016). Association of Borderline Pulmonary Hypertension With Mortality and Hospitalization in a Large Patient Cohort: Insights From the Veterans Affairs Clinical Assessment, Reporting, and Tracking Program. Circulation.

[11] Douschan P., Kovacs G., Avian A., Foris V., Gruber F., Olschewski A., Olschewski H. (2018). Mild Elevation of Pulmonary Arterial Pressure as a Predictor of Mortality. Am. J. Respir. Crit. Care Med..

[12] Kolte D., Lakshmanan S., Jankowich M.D., Brittain E.L., Maron B.A., Choudhary G. (2018). Mild Pulmonary Hypertension Is Associated With Increased Mortality: A Systematic Review and Meta-Analysis. J. Am. Heart Assoc..

[13] Pabst S., Hammerstingl C., Grau N., Kreuz J., Grohe C., Juergens U.R., Nickenig G., Skowasch D. (2013). Pulmonary arterial hypertension in patients with sarcoidosis: The Pulsar single center experience. Adv. Exp. Med. Biol..

[14] Alhamad E.H., Idrees M.M., Alanezi M.O., Alboukai A.A., Shaik S.A. (2010). Sarcoidosis-associated pulmonary hypertension: Clinical features and outcomes in Arab patients. Ann. Thorac. Med..

[15] Huitema M.P., Bakker A.L.M., Mager J.J., Rensing B., Smits F., Snijder R.J., Grutters J.C., Post M.C. (2019). Prevalence of pulmonary hypertension in pulmonary sarcoidosis: The first large European prospective study. Eur. Respir. J..

[16] Zhang S., Tong X., Zhang T., Wang D., Liu S., Wang L., Fan H. (2021). Prevalence of Sarcoidosis-Associated Pulmonary Hypertension: A Systematic Review and Meta-Analysis. Front. Cardiovasc. Med..

[17] Shorr A.F., Helman D.L., Davies D.B., Nathan S.D. (2005). Pulmonary hypertension in advanced sarcoidosis: Epidemiology and clinical characteristics. Eur. Respir. J..

[18] Baughman R.P., Shlobin O.A., Wells A.U., Alhamad E.H., Culver D.A., Barney J., Cordova F.C., Carmona E.M., Scholand M.B., Wijsenbeek M. (2018). Clinical features of sarcoidosis associated pulmonary hypertension: Results of a multi-national registry. Respir. Med..

[19] Islam M.I., Eggert M., Bernens M., Sill J. (2020). Pulmonary veno-occlusive disease in sarcoidosis patient: A rare entity causing pulmonary hypertension. Chest.

[20] Nunes H., Humbert M., Capron F., Brauner M., Sitbon O., Battesti J.P., Simonneau G., Valeyre D. (2006). Pulmonary hypertension associated with sarcoidosis: Mechanisms, haemodynamics and prognosis. Thorax.

[21] Ungprasert P., Srivali N., Wijarnpreecha K., Thongprayoon C. (2015). Sarcoidosis and risk of venous thromboembolism: A systematic review and meta-analysis. Sarcoidosis Vasc. Diffus. Lung Dis..

[22] Condado J.F., Babaliaros V., Henry T.S., Kaebnick B., Kim D., Staton G.W. (2016). Pulmonary stenting for the treatment of sarcoid induced pulmonary vascular stenosis. Sarcoidosis Vasc. Diffus. Lung Dis..

[23] Damuth T.E., Bower J.S., Cho K., Dantzker D.R. (1980). Major pulmonary artery stenosis causing pulmonary hypertension in sarcoidosis. Chest.

[24] Hamilton-Craig C.R., Slaughter R., McNeil K., Kermeen F., Walters D.L. (2009). Improvement after angioplasty and stenting of pulmonary arteries due to sarcoid mediastinal fibrosis. Heart Lung Circ..

[25] Hoffstein V., Ranganathan N., Mullen J.B. (1986). Sarcoidosis simulating pulmonary veno-occlusive disease. Am. Rev. Respir. Dis..

[26] Takemura T., Matsui Y., Saiki S., Mikami R. (1992). Pulmonary vascular involvement in sarcoidosis: A report of 40 autopsy cases. Hum. Pathol..

[27] Lal C., Medarov B.I., Judson M.A. (2015). Interrelationship between sleep-disordered breathing and sarcoidosis. Chest.

[28] Sonnweber T., Pizzini A., Tancevski I., Löffler-Ragg J., Weiss G. (2020). Anaemia, iron homeostasis and pulmonary hypertension: A review. Intern. Emerg. Med..

[29] Nickel N.P., Galura G.M., Zuckerman M.J., Hakim M.N., Alkhateeb H., Mukherjee D., Austin E.D., Heresi G.A. (2021). Liver abnormalities in pulmonary arterial hypertension. Pulm. Circ..

[30] Shlobin O.A., Kouranos V., Barnett S.D., Alhamad E.H., Culver D.A., Barney J., Cordova F.C., Carmona E.M., Scholand M.B., Wijsenbeek M. (2020). Physiological predictors of survival in patients with sarcoidosis-associated pulmonary hypertension: Results from an international registry. Eur. Respir. J..

[31] Kouranos V., Sharma R. (2021). Cardiac sarcoidosis: State-of-the-art review. Heart.

[32] Guazzi M., Borlaug B.A. (2012). Pulmonary hypertension due to left heart disease. Circulation.

[33] Baughman R.P., Engel P.J., Taylor L., Lower E.E. (2010). Survival in sarcoidosis-associated pulmonary hypertension: The importance of hemodynamic evaluation. Chest.

[34] Patel M.B., Mor-Avi V., Murtagh G., Bonham C.A., Laffin L.J., Hogarth D.K., Medvedofsky D., Lang R.M., Patel A.R. (2016). Right Heart Involvement in Patients with Sarcoidosis. Echocardiography.

[35] Swigris J.J., Olson A.L., Huie T.J., Fernandez-Perez E.R., Solomon J.J., Sprunger D., Brown K.K. (2011). Increased risk of pulmonary embolism among US decedents with sarcoidosis from 1988 to 2007. Chest.

[36] Ungprasert P., Crowson C.S., Matteson E.L. (2017). Association of Sarcoidosis With Increased Risk of VTE: A Population-Based Study, 1976 to 2013. Chest.

[37] Crawshaw A.P., Wotton C.J., Yeates D.G., Goldacre M.J., Ho L.P. (2011). Evidence for association between sarcoidosis and pulmonary embolism from 35-year record linkage study. Thorax.

[38] Boucly A., Cottin V., Nunes H., Jaïs X., Tazi A., Prévôt G., Reynaud-Gaubert M., Dromer C., Viacroze C., Horeau-Langlard D. (2017). Management and long-term outcomes of sarcoidosis-associated pulmonary hypertension. Eur. Respir. J..

[39] Savale L., Huitema M., Shlobin O., Kouranos V., Nathan S.D., Nunes H., Gupta R., Grutters J.C., Culver D.A., Post M.C. (2022). WASOG statement on the diagnosis and management of sarcoidosis-associated pulmonary hypertension. Eur. Respir. Rev..

[40] Ng C.S., Wells A.U., Padley S.P. (1999). A CT sign of chronic pulmonary arterial hypertension: The ratio of main pulmonary artery to aortic diameter. J. Thorac. Imaging.

[41] Huitema M.P., Spee M., Vorselaars V.M., Boerman S., Snijder R.J., van Es H.W., Reesink H.J., Grutters J.C., Post M.C. (2016). Pulmonary artery diameter to predict pulmonary hypertension in pulmonary sarcoidosis. Eur. Respir. J..

[42] Stein P.D., Fowler S.E., Goodman L.R., Gottschalk A., Hales C.A., Hull R.D., Leeper K.V., Popovich J., Quinn D.A., Sos T.A. (2006). Multidetector computed tomography for acute pulmonary embolism. N. Engl. J. Med..

[43] Bourbonnais J.M., Samavati L. (2008). Clinical predictors of pulmonary hypertension in sarcoidosis. Eur. Respir. J..

[44] Mirsaeidi M., Omar H.R., Baughman R., Machado R., Sweiss N. (2016). The association between BNP, 6MWD test, DLCO% and pulmonary hypertension in sarcoidosis. Sarcoidosis Vasc. Diffus. Lung Dis..

[45] Gupta R., Baughman R.P., Nathan S.D., Wells A.U., Kouranos V., Alhamad E.H., Culver D.A., Barney J., Carmona E.M., Cordova F.C. (2022). The six-minute walk test in sarcoidosis associated pulmonary hypertension: Results from an international registry. Respir. Med..

[46] Tandon R., Baughman R.P., Stanley J., Khan A.A. (2017). The link between chronic thromboembolic pulmonary hypertension and sarcoidosis: Association or visual masquerade?. Sarcoidosis Vasc. Diffus. Lung Dis..

[47] Kelle S., Bucciarelli-Ducci C., Judd R.M., Kwong R.Y., Simonetti O., Plein S., Raimondi F., Weinsaft J.W., Wong T.C., Carr J. (2020). Society for Cardiovascular Magnetic Resonance (SCMR) recommended CMR protocols for scanning patients with active or convalescent phase COVID-19 infection. J. Cardiovasc. Magn. Reson..

[48] Ahmadi A., Ohira H., Mielniczuk L.M. (2015). FDG PET imaging for identifying pulmonary hypertension and right heart failure. Curr. Cardiol. Rep..

[49] Hagan G., Southwood M., Treacy C., Ross R.M., Soon E., Coulson J., Sheares K., Screaton N., Pepke-Zaba J., Morrell N.W. (2011). ^18^FDG PET imaging can quantify increased cellular metabolism in pulmonary arterial hypertension: A proof-of-principle study. Pulm. Circ..

[50] Marsboom G., Wietholt C., Haney C.R., Toth P.T., Ryan J.J., Morrow E., Thenappan T., Bache-Wiig P., Piao L., Paul J. (2012). Lung ^18^F-fluorodeoxyglucose positron emission tomography for diagnosis and monitoring of pulmonary arterial hypertension. Am. J. Respir. Crit. Care Med..

[51] Maier A., Liao S.L., Lescure T., Robson P.M., Hirata N., Sartori S., Narula N., Vergani V., Soultanidis G., Morgenthau A. (2022). Pulmonary Artery ^18^F-Fluorodeoxyglucose Uptake by PET/CMR as a Marker of Pulmonary Hypertension in Sarcoidosis. JACC Cardiovasc. Imaging.

[52] Zhao L., Ashek A., Wang L., Fang W., Dabral S., Dubois O., Cupitt J., Pullamsetti S.S., Cotroneo E., Jones H. (2013). Heterogeneity in lung ^18^FDG uptake in pulmonary arterial hypertension: Potential of dynamic ^18^FDG positron emission tomography with kinetic analysis as a bridging biomarker for pulmonary vascular remodeling targeted treatments. Circulation.

[53] Ovadia M. (2022). Pulmonary Arterial ^18^F-FDG Uptake in Sarcoidosis: A Novel Biosignal for Subclinical Pulmonary Hypertension. JACC Cardiovasc. Imaging.

[54] Milman N., Svendsen C.B., Iversen M., Videbaek R., Carlsen J. (2009). Sarcoidosis-associated pulmonary hypertension: Acute vasoresponsiveness to inhaled nitric oxide and the relation to long-term effect of sildenafil. Clin. Respir. J..

[55] Preston I.R., Klinger J.R., Landzberg M.J., Houtchens J., Nelson D., Hill N.S. (2001). Vasoresponsiveness of sarcoidosis-associated pulmonary hypertension. Chest.

[56] daSilva-deAbreu A., Mandras S.A. (2021). Sarcoidosis-Associated Pulmonary Hypertension: An Updated Review and Discussion of the Clinical Conundrum. Curr. Probl. Cardiol..

[57] Huitema M.P., Mathijssen H., Mager J.J., Snijder R.J., Grutters J.C., Post M.C. (2020). Sarcoidosis-Associated Pulmonary Hypertension. Semin. Respir. Crit. Care Med..

[58] Cullivan S., Gaine S., Sitbon O. (2023). New trends in pulmonary hypertension. Eur. Respir. Rev..

[59] Macera F., Vachiéry J.L. (2021). Management of Pulmonary Hypertension in Left Heart Disease. Methodist. DeBakey Cardiovasc. J..

[60] Le Pavec J., Valeyre D., Gazengel P., Holm A.M., Schultz H.H., Perch M., Le Borgne A., Reynaud-Gaubert M., Knoop C., Godinas L. (2021). Lung transplantation for sarcoidosis: Outcome and prognostic factors. Eur. Respir. J..

[61] Weill D., Benden C., Corris P.A., Dark J.H., Davis R.D., Keshavjee S., Lederer D.J., Mulligan M.J., Patterson G.A., Singer L.G. (2015). A consensus document for the selection of lung transplant candidates: 2014--an update from the Pulmonary Transplantation Council of the International Society for Heart and Lung Transplantation. J. Heart Lung Transplant..

[62] Fadel E., Mercier O., Mussot S., Leroy-Ladurie F., Cerrina J., Chapelier A., Simonneau G., Dartevelle P. (2010). Long-term outcome of double-lung and heart-lung transplantation for pulmonary hypertension: A comparative retrospective study of 219 patients. Eur. J. Cardio-Thorac. Surg..

[63] Shah L. (2007). Lung Transplantation in Sarcoidosis. Semin. Respir. Crit. Care Med..

[64] Wryobeck J.M., Lippo G., McLaughlin V., Riba M., Rubenfire M. (2007). Psychosocial aspects of pulmonary hypertension: A review. Psychosomatics.

[65] Bussotti M., Sommaruga M. (2018). Anxiety and depression in patients with pulmonary hypertension: Impact and management challenges. Vasc. Health Risk Manag..

[66] Rawlings G.H., Novakova B., Armstrong I., Thompson A.R. (2023). A systematic review of psychological interventions in adults with pulmonary hypertension: Is the evidence-base disproportionate to the problem?. Clin. Respir. J..

[67] Currie B.M., Davies E.W., Beaudet A., Stassek L., Kleinman L., Baughman R.P. (2021). Symptoms, impacts, and suitability of the Pulmonary Arterial Hypertension-Symptoms and Impact (PAH-SYMPACT™) questionnaire in patients with sarcoidosis-associated pulmonary hypertension (SAPH): A qualitative interview study. BMC Pulm. Med..

[68] Chin K.M., Gomberg-Maitland M., Channick R.N., Cuttica M.J., Fischer A., Frantz R.P., Hunsche E., Kleinman L., McConnell J.W., McLaughlin V.V. (2018). Psychometric Validation of the Pulmonary Arterial Hypertension-Symptoms and Impact (PAH-SYMPACT) Questionnaire: Results of the SYMPHONY Trial. Chest.

[69] Baughman R.P., Culver D.A., Cordova F.C., Padilla M., Gibson K.F., Lower E.E., Engel P.J. (2014). Bosentan for Sarcoidosis-Associated Pulmonary Hypertension: A Double-Blind Placebo Controlled Randomized Trial. Chest.

[70] Judson M.A., Highland K.B., Kwon S., Donohue J.F., Aris R., Craft N., Burt S., Ford H.J. (2011). Ambrisentan for sarcoidosis associated pulmonary hypertension. Sarcoidosis Vasc. Diffus. Lung Dis..

[71] Mathijssen H., Huitema M.P., Bakker A.L.M., Mager J.J., Snijder R.J., Grutters J.C., Post M.C. (2020). Safety of macitentan in sarcoidosis-associated pulmonary hypertension: A case-series. Sarcoidosis Vasc. Diffus. Lung Dis..

[72] Milman N., Burton C.M., Iversen M., Videbaek R., Jensen C.V., Carlsen J. (2008). Pulmonary hypertension in end-stage pulmonary sarcoidosis: Therapeutic effect of sildenafil?. J. Heart Lung Transplant..

[73] Ford H.J., Baughman R.P., Aris R., Engel P., Donohue J.F. (2016). Tadalafil therapy for sarcoidosis-associated pulmonary hypertension. Pulm. Circ..

[74] Keir G.J., Walsh S.L., Gatzoulis M.A., Marino P.S., Dimopoulos K., Alonso R., Raposeiras-Roubin S., Renzoni E.A., Maher T.M., Wells A.U. (2014). Treatment of sarcoidosis-associated pulmonary hypertension: A single centre retrospective experience using targeted therapies. Sarcoidosis Vasc. Diffus. Lung Dis..

[75] Baughman R.P., Shlobin O.A., Gupta R., Engel P.J., Stewart J.I., Lower E.E., Rahaghi F.F., Zeigler J., Nathan S.D. (2022). Riociguat for Sarcoidosis-Associated Pulmonary Hypertension: Results of a 1-Year Double-Blind, Placebo-Controlled Trial. Chest.

[76] Fisher K.A., Serlin D.M., Wilson K.C., Walter R.E., Berman J.S., Farber H.W. (2006). Sarcoidosis-associated pulmonary hypertension: Outcome with long-term epoprostenol treatment. Chest.

[77] Baughman R.P., Judson M.A., Lower E.E., Highland K., Kwon S., Craft N., Engel P.J. (2009). Inhaled iloprost for sarcoidosis associated pulmonary hypertension. Sarcoidosis Vasc. Diffus. Lung Dis..

[78] Abston E., Hon S., Lawrence R., Berman J., Govender P., Farber H.W. (2020). Treatment of newly diagnosed sarcoid-associated pulmonary hypertension with ambrisentan and tadalafil combination therapy. Sarcoidosis Vasc. Diffus. Lung Dis..

[79] Sumimoto K., Taniguchi Y., Emoto N., Hirata K.I. (2021). Combination therapy with pulmonary arterial hypertension targeted drugs and immunosuppression can be a useful strategy for sarcoidosis-associated pulmonary hypertension: A case report. Eur. Heart J. Case Rep..

